# Quality of Life of People Living with Dementia and Care Partners in 3 Countries: Canada, The Netherlands and Poland

**DOI:** 10.1002/alz.095675

**Published:** 2025-01-09

**Authors:** Marwa Ilali

**Affiliations:** ^1^ McGill University, Montreal, QC Canada

## Abstract

The global prevalence of dementia is on a concerning rise, currently affecting 55 million people. It is projected to triple in the next 30 years, leading to a profound impact on the quality of life (QoL) for both people living with dementia (PLWDs) and care partners (CPs). The compounded challenges, encompassing mental, physical, and financial aspects, significantly affect the QoL of PLWDs and CPs, creating an urgent need for comprehensive understanding and tailored interventions. Beyond medical considerations, ethical principles highlight respecting the autonomy and dignity of PLWDs, requiring a delicate balance in meeting their care needs while minimizing CPs' burden. Long‐term care planning is imperative at both individual and societal levels, necessitating sustainable healthcare policies and supportive environments. Existing healthcare and social care responses have limitations, underlining the need for rigorous scientific inquiry to enhance understanding, address unique needs, and improve QoL. The purpose of this research is to explore the multifaceted aspects of improving QoL for PLWDs and CPs in Canada, the Netherlands, and Poland. It seeks to identify predictors, understand experiences, and propose recommendations to improve QoL in the three countries. To address these objectives, the research questions are: What are the predictors of QoL among PLWDs and CPs in Canada, the Netherlands, and Poland? How do PLWDs and CPs experience dementia in these diverse regions? What recommendations can be identified to enhance the QoL of PLWDs and CPs? Employing a results‐based convergent synthesis design approach, this research involves conducting a cross‐sectional survey utilizing random forest models to determine predictors of QoL. Semi‐structured interviews and focus groups with PLWDs and CPs explore their experiences with living with dementia. Deliberative dialogues with diverse stakeholders, including PLWDs, CPs, primary care providers, and policymakers, are facilitated to generate recommendations. To contribute actionable knowledge, evidence‐based recommendations, and support frameworks to enhance the QoL of PLWDs and CPs.

